# Resolution of Periodic Breathing in a Child with Idiopathic Pulmonary Arterial Hypertension

**DOI:** 10.1155/2017/3280572

**Published:** 2017-08-23

**Authors:** Saadoun Bin-Hasan, Abdullah Khayat, Tilman Humpl, Janette T. Reyes, Suhail Al-Saleh

**Affiliations:** ^1^Department of Pediatrics, University of Toronto, Toronto, ON, Canada; ^2^Department of Pediatrics, Division of Respiratory Medicine, The Hospital for Sick Children, Toronto, ON, Canada; ^3^Department of Pediatrics, Division of Cardiology, The Hospital for Sick Children, Toronto, ON, Canada

## Abstract

Central sleep apnea (CSA) and periodic breathing are unusual findings described in pediatric patients with congestive heart failure. However, CSA has not been reported in children with pulmonary hypertension. We hereby report on a 10-year-old girl with idiopathic pulmonary arterial hypertension who had frequent central events in a periodic breathing fashion seen in her polysomnography, which was normalized following medical treatment leading to improvement of the pulmonary pressures.

## 1. Introduction

Idiopathic pulmonary arterial hypertension (IPAH) is an uncommon disorder in childhood with an incidence of 0.48 cases per million children per year and a prevalence of 2.1 cases per million, based on the United Kingdom registry [1]. If left untreated, the median reported survival rate is 10 months in the pediatric population compared to 2.8 years in adults [2].

Central sleep apnea (CSA) and periodic breathing (PB) have occasionally been described in adults with pulmonary arterial hypertension (PAH) [3]. Cheyne-Stokes respiration (CSR) on the other hand, which is a form of PB, is more commonly seen in patients with congestive heart failure (CHF), and it has been considered to be a poor prognostic factor as it is associated with higher morbidity and mortality [4, 5].

We describe in this report a child with IPAH who had evidence of PB on an overnight in-lab polysomnography (PSG) which was normalized following the improvement of the pulmonary pressures.

To our knowledge, this is the first report describing a complete resolution of PB in a child with IPAH after optimal pharmacological management.

## 2. Report of Case

**Figure 1:**
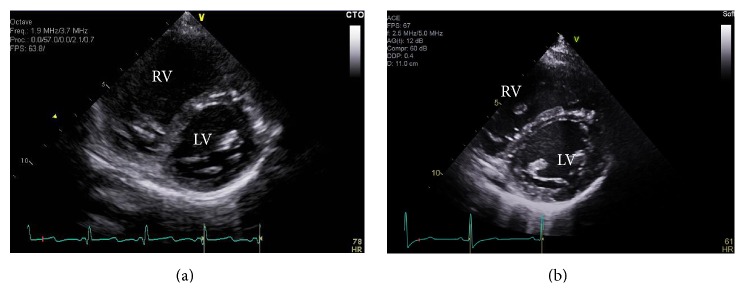
(a) ECHO at diagnosis showing a short access view of the ventricles at systole with interventricular septal thickening and flattening; LV: left ventricle; RV: right ventricle. (b) At follow-up (after treatment); significant improvement in the interventricular septal morphology.

The patient, a 10-year-old previously healthy female, presented with a progressive decline in her exercise tolerance and episodes of shortness of breath with activity as well as rest and presyncopal events over 3-4-month period. Her symptoms were New York Heart Association (NYHA) functional class III, and she had a low exercise capacity with 380 m total distance achieved in the 6-minute walk test (6MWT). She was diagnosed with IPAH after a thorough work-up. The transthoracic echocardiogram (ECHO) showed evidence of significant pulmonary hypertension with a right ventricular systolic pressure (RVSP) estimated to be suprasystemic, dilated right ventricle (RV), septal flattening in systole, and diastole with dilated main pulmonary artery (MPA) and excluded the presence of congenital heart disease (Figure 1(a)). The electrocardiogram (ECG) showed normal sinus rhythm with a heart rate of 84 beats/min and a right atrial enlargement. The NT-pro BNP was 147 pmol/L. The computed tomography (CT) excluded the presence of a parenchymal lung disease, and the liver ultrasound showed no evidence of portal hypertension. The ventilation-perfusion scan (V/Q scan) showed no significant V/Q mismatch, and the pulmonary function test revealed normal flows and volumes.

**Figure 2:**
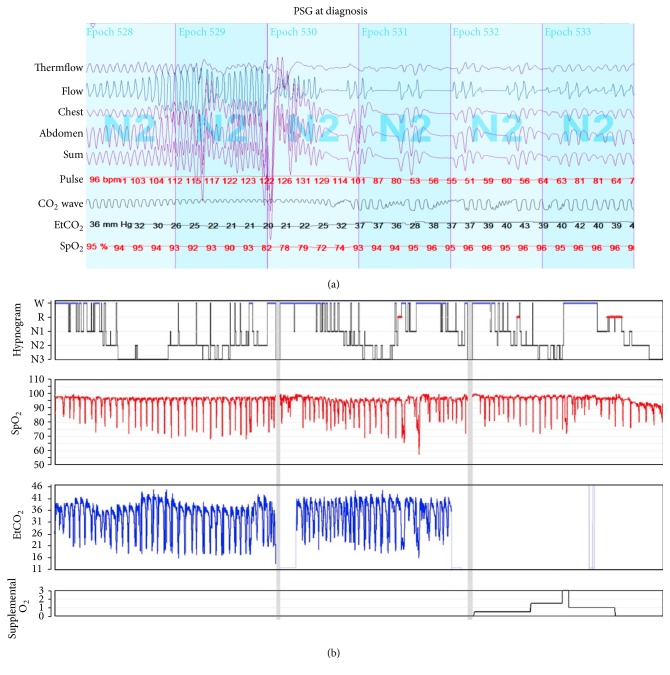
(a) 3-minute polysomnographical recording showing the periodic breathing pattern. (b) Hypnogram from the entire night illustrating the sleep stages; W: wakefulness, R: REM, N1–N3: non-REM sleep (stage 1–3), SpO_2_: oxygen saturation, and EtCO_2_: end tidal carbon dioxide. Supplemental oxygen was added during the last third of the night.

She had a PSG to assess for sleep disordered breathing explicitly ruling out obstructive sleep apnea as a potential cause of PH [6]. PSG baseline segment revealed significant sleep fragmentation with increased awakenings and arousals (arousal index = 23.1/hr). Frequent respiratory episodes with a similar unique pattern were seen. Each started with a decrease in the baseline SpO_2_ followed by a rapid transient increase in the respiratory rate (up to 38 breaths per minute) for about 30–90 seconds, leading to a drop in the baseline etCO_2_ (20–22 mmHg). Those events were terminated by central events occurring in a PB fashion (Figure 2). The overall central apnea hypopnea index (CAHI) was 6.3/hour and the PB was 36.6 minutes (10.8%) of the total sleep time.

Given the frequency of the central events as well as the severity of the SpO_2_ desaturation, supplemental oxygen was trialed. Although the frequency of the SpO_2_ desaturation and PB events did not significantly change, the degree of desaturations improved from 57 to 72% with an average baseline SpO_2_ saturation of 97% from 93% off oxygen (CAHI = 5.7 while on supplemental oxygen). After the sleep study, she was discharged home on 3 liters/minute of oxygen via nasal prongs during sleep. Further neurological evaluation after the sleep study including a brain MRI and EEG was within normal limits and excluded any neurological reason for the frequent central events.

**Figure 3:**
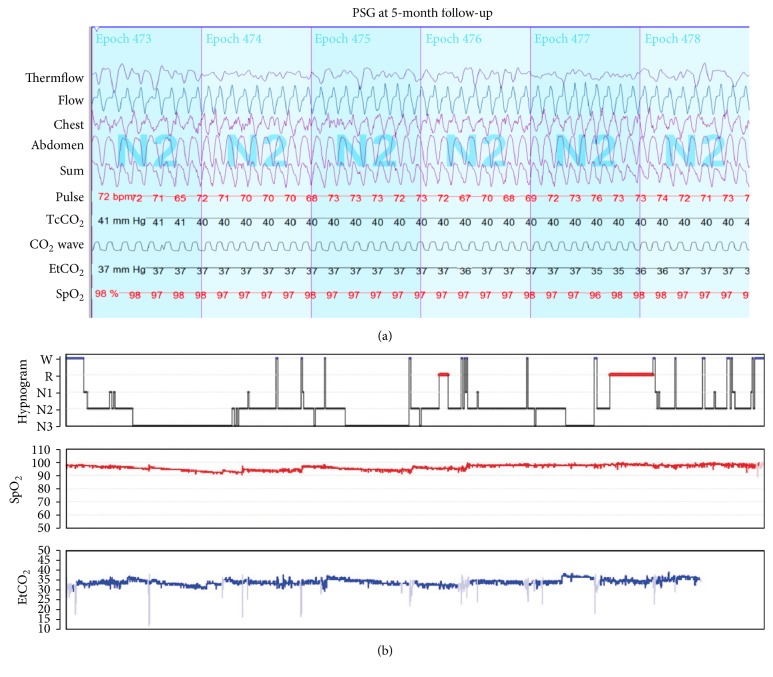
(a) 3-minute polysomnographical recording showing the normalization of the breathing pattern. (b) Hypnogram from the entire night illustrating the sleep stages; W: wakefulness, R: REM, N1–N3: non-REM sleep (stage 1–3), SpO_2_: oxygen saturation, and EtCO_2_: end tidal carbon dioxide.

The planned right heart cardiac catheterization was deemed not safe to pursue at the time of the diagnosis given the hemodynamic instability. Thus, treprostinil, a synthetic analog of prostacyclin (PGI_2_) therapy, was empirically initiated as a continuous subcutaneous infusion. At 5-month follow-up, 6MWT increased to 660 m and the patient was in NYHA class I. The ECHO showed a significant reduction in the pulmonary pressures from previous and estimated at <1/2 systemic RVSP as the septum was mildly flat in systole, RVSP (TR) 50 mmHg (Figure 1(b)). The NT-pro BNP was 12 pmol/L. She had a normal sinus rhythm in the ECG with right atrial enlargement. The PSG reflected a normalization of her breathing pattern with no evidence of PB (Figure 3). The baseline mean SpO_2_ improved to 95% and there were significantly fewer desaturations throughout the night (desaturation index = 0.5/hr). The CAHI was within normal limits at 0.5/hr (Table 1).

## 3. Discussion

PB during sleep is characterized by periods of central pauses in respiration separated by normal breathing pattern [7]. It could be classified as physiological, as seen in preterm infants relating to the immaturity of the breathing centers, or pathological [8]. CSR is a form of PB characterized by a prolonged crescendo-decrescendo pattern of respiration, followed by a central event, either apnea or hypopnea [3]. PB in the form of CSR has been reported in up to 50% of adult patients with congestive heart failure (CHF), and its presence heralds a worse prognosis [5].

The mechanism of PB and CSA in IPAH has not yet been fully understood; however, in our case it might be similar to that described in CHF. First, the cardiac output will likely be reduced leading to a prolonged circulation time, thus hypoxemia. Second, the hypoxemic milieu would stimulate the peripheral chemoreceptors leading to hyperventilation and this change in ventilation would drive the PaCO_2_ below the apnea threshold giving rise to central events [3].

Few studies have reported the prevalence of central sleep apnea and PB in adult patients with PAH [4, 5, 9]. Pitsiou et al. [4] reported on a 46-year-old woman with IPAH and PSG features of PB. The patient was treated with bosentan, and oral anticoagulation therapy and a follow-up PSG showed a complete resolution of the PB. In pediatrics, data is even more limited, with only one reported case of CSR in a boy with PAH and Trisomy 21 [10]. However, the presence of the PAH leading to CSR was related to the atrioventricular septal defect as explained by the authors. To our knowledge, this is the first report of PB in pediatric IPAH with a complete resolution after optimal pharmaceutical treatment.

## 4. Conclusion

This case supports the importance of PSG in pediatric patients with IPAH, not only to exclude OSA as a potential cause but also to assess for the presence of PB. We also show that the presence of PB might be a sign of disease severity and can be a marker of response to medical treatment. Prompt diagnoses and management of the IPAH would improve SDB in this vulnerable population and might lead to a favorable prognosis. However, more studies are needed to evaluate the extent of PB in children with IPAH.

## Figures and Tables

**Table 1:** PSG results of the patient before and after treprostinil and oxygen therapy.

Results	At diagnosis	After treprostinil
TST, min	338	269.5
Sleep latency, min	11.4	6.4
Sleep efficiency, %	67.3	70.8
REM latency, min	276.5	136.5
Stage N1, %TST	24.9	7.4
Stage N2, %TST	46	57
Stage N3, %TST	24.4	28.2
REM, %TST	4.7	7.4
Arousals, total index	23.1	9.1
Mean sleep SpO_2_ (%)	93	95
Minimum SpO_2_ (%)	57	90
Desaturation Index (events/hr)	15.7	0.5
Highest TcCO_2_/etCO_2_ (mmHg)	45	46
Mean TcCO_2_/etCO_2_ (mmHg)	32	39
OAHI (events/hour)	0	0
CAHI (events/hour)	6.3	0.5
RVSP (estimated)	Suprasystemic	<1/2 systemic
NT-proBNP (pmol/L)	147	12
6MWT (meters)	380	660
Mean SpO_2_ during wakefulness (%)	94	99
Mean TcCO_2_/etCO_2_ during wakefulness (%)	30	35

TST: total sleep time, REM: rapid eye movements, SaO_2_: oxygen saturation, TcCO_2_: transcutaneous carbon dioxide, etCO_2_: end tidal carbon dioxide, OAHI: obstructive apnea-hypopnea index, CAHI: central apnea-hypopnea index, RVSP: right ventricular systolic pressure, NT-proBNP: N-terminal pro b-type natriuretic peptide, and6MWT: 6-minute walk test.
